# Nutritional status of tuberculosis patients, a comparative cross-sectional study

**DOI:** 10.1186/s12890-019-0953-0

**Published:** 2019-10-21

**Authors:** Berhanu Elfu Feleke, Teferi Elfu Feleke, Fantahun Biadglegne

**Affiliations:** 1 Department of Epidemiology and Biostatistics, University of Bahir Dar, Bahir Dar, Ethiopia; 2 Department of pediatrics, University of St Paul, Addis Ababa, Ethiopia; 3 Department of medical laboratory sciences, University of Bahir Dar, Bahir Dar, Ethiopia

**Keywords:** Malnutrition, Tuberculosis, Predictors, Ethiopia

## Abstract

**Background:**

Each year, more than 13.7 million people became an active case of tuberculosis and more than 1.5 million cases of TB patient will die. The association between TB and malnutrition is bi-directional, TB leads the patient to malnutrition, and malnutrition increases the risk of developing active TB by 6 to 10 times. Improving the nutrition of individual greatly reduces tuberculosis. The aims of this study were to assess the nutritional status and determinants of underweight among TB patients.

**Methods:**

A comparative cross-sectional study design was implemented. The sample size was calculated using 95% CI, 90% power, the prevalence of malnutrition in TB patients 50%,

TB patients to TB free resident ratio of 3, the design effect of 2 and a 5% non-response rate. Systematic random sampling was used to select TB patients and simple random sampling technique was used to select TB free residents. The data were collected from July 2015–May 2018. The data were collected by interviewing the patient, measuring anthropometric indicators and collecting the stool and blood samples. The data were entered into the computer using Epi-info software and analyzed using SPSS software. Descriptive statistics were used to find the proportion of malnutrition. Binary logistic regression was used to identify the determinants of malnutrition.

**Results:**

A total of 5045 study participants (1681 TB patients and 3364 TB free residents) were included giving for the response rate of 93.1%. The prevalence of underweight among TB patients was 57.17% (95% CI: 54.80, − 59.54%) and 88.52% of TB patients were anemic. The prevalence of malnutrition (underweight) among TB free residents was 23.37% (95% CI: 21.93–24.80). The nutritional status of TB patients was determined by site of infection AOR: 0.68 [0.49–0.94], sex of the patient AOR: 0.39 [0.25–0.56], residence AOR: 3.84 [2.74–5.54], intestinal parasite infection AOR: 7 [5.2–9.95], problematic alcohol use AOR: 1.52 [1.17–2.13].

**Conclusion:**

High proportions of TB patients were malnourished. TB patients were highly susceptible to malnutrition and even a very distal reason for malnutrition in the community became a proximal cause for TB patients.

## Background

Underweight is a malnutrition stage in which the body mass index (BMI) of adult scores less than 18.5 KG/M^2^ cuts-points [1, 2]. It results from an imbalance between the supply of food and the body demands for the nutrients. Conditions like genetics, metabolism disorders, medication side effects, eating disorders, and tuberculosis predispose to underweight [3, 4].

Tuberculosis (TB) is an infectious disease caused by mycobacterium TB species. According to the world health organization (WHO), tuberculosis attacks 10 million people and kills 1.3 million people every year [5]. The association between TB and malnutrition is bi-directional, TB predisposes the patient to malnutrition and malnutrition increases the risk of developing active TB by 6 to 10 folds [6–11]. One-quarter of TB in the world was as a result of malnutrition, improving the nutritional status of the individual decreases the risk of TB [12]. Additionally, malnutrition increases TB relapse and mortality [13–20].

The treatment outcomes of TB patients can be improved by studying their nutritional status [13, 20]. Incorporating nutritional support during directly observed treatment strategy (DOTS) increases the probability of favorable treatment outcomes [16, 19, 21–27].

The prevalence of malnutrition in the South American continent range from 20 - 50% [28, 29], in the Asian continent malnutrition among TB patient ranges from 68.6 - 87% [11, 20] and in the United Kingdom, the body mass index of TB patients was 13% lower than the general community [18]. On the African continent, 29 - 61% of TB patients were malnourished [14, 24, 30–33].

The tuberculosis treatment guideline neglects the nutritional supplementation part of an intervention. Priority was not given by decision-makers as a result of evidence scarcity. This study will generate baseline information to give priority to the nutritional intervention part of TB treatment.

The objectives of this research were to estimate and compare the proportion of underweight and their determinants among the TB patients in a resource-limited setting.

## Methods

A comparative cross-sectional study design was implemented. This study was conducted in Amhara regional state of Ethiopia. The region contains 25,000,000 population and every year more than 25,000 tuberculosis cases are reported from the health facilities. The target population was TB patients receiving the DOTS in the health centers of the Amhara region. New TB patients diagnosed during the data collection method were included. TB patients not willing to participate during the study and TB patients unable to communicate properly were excluded. The nutritional status of TB patients was compared with TB free residents. The sample size was calculated using Epi-info software version 7 assuming 95% CI, 90% power, the proportion of malnutrition in TB patients 50%, TB patients to TB free resident ratio of 3, the design effect of 2 and a 5% non-response rate [6, 34]. Finally, the estimated sample size was 1806 TB patients and 3612 TB free residents. The systematic random sampling technique was used to select TB patients from the selected governmental health facilities. Ethiopia adopts the tree health tier system; primary level health care (including the health center and district hospital and expected to serve up to 100,000 population), secondary level health care (includes the general hospital and expected to serve up to 1.5 million population) and the tertiary level health care (includes the specialized referral hospitals and expected to serve up to 5 million people). TB free residents were selected randomly from the community from the nearby house of the TB patients. The data were collected from July 2015–May 2018.

The data were collected using patient interview, measuring anthropometric indicators and collecting the stool and blood samples. The stool samples were collected to adjust for the effect of intestinal parasites on nutritional status because, in resource-limited settings like Ethiopia, intestinal parasites are the main predictor’s malnutrition. The blood samples were collected to see the effect of anemia on underweight because literature reports their relationship. For the patient interview part, the questionnaire was prepared in English then translated to Amharic (local language) then back to English to keep its consistency. The interview was conducted by 30 diploma nurse professionals and supervised by 8 degree holder health professionals. Weight and height of each study participant were measured by clinical nurses. Digital weight scale was used to measure the weight of each study participant and weight was measured to the nearest 0.1 kg. Height was measured using the vertical measuring rod to the nearest 0.1 cm. The participants’ shoes, hair clips, and braids were removed during the measurement. Participants were positioned feet together, feet flat on the ground, heels touching the backplate of the measuring instrument, legs straight, buttocks against the backboard, scapula against the backboard and arms were loosely at their side [1, 35].

The blood and stool samples were collected by 13-degree holder laboratory technologists and supervised by second-degree holder laboratory technologists. For each study participant, one gram stool sample was collected in 10 ml SAF (sodium acetate-acetic acid-formalin solution) and transported with a preservative to the regional laboratory. A concentration technique was used. The stool samples were well mixed and filtered using a funnel with gauze, then centrifuged for one minute at 2000 RPM (revolution per minute) and the supernatant was discarded. 7 ML (Milliliter) normal saline was added, mixed with a wooden stick, 3 ML ether was added and mixed well then centrifuged for 5 min at 2000 RPM. Finally, the supernatant was discarded and the whole sediment was examined for parasites. 5 ML blood sample was collected from a study participant following standard operating procedures to measure the hemoglobin concentration and the red cell indices of patients using Mindray hematology analyzer [36]. CAGE tool was used to assess the problematic alcohol use. Study participants unable to read and wrote were labeled as illiterate.

To maintain the quality of the data pretest was conducted, training was given for data collectors and supervisors. The whole data collection processes were closely supervised by the investigators and supervisors.

The data were entered in the computer using Epi-info software transferred into SPSS software version 20 and WHO Anthro-plus software for the analysis. Descriptive statistics were used to identify the proportion of malnutrition. Binary logistic regression was used to identify the determinants of malnutrition. Variables with a *p*-value less than 0.05 were declared as predictors of malnutrition. Body mass index (BMI) was used to assess nutritional status. BMI was computed as = weight (in kilogram)/ (height in meter) ^2^. BMI less than 18.5 KG/M^2^ was considered underweight [1].

Ethical clearance was obtained from the University of Bahir Dar ethical review committee. Formal permission was obtained from the Amhara National Regional State Health Bureau ethics committee and the respective authorities. Written informed consent was obtained from each study participant. The confidentiality of the data was kept at all steps. Study participants the right to withdraw from the study at any points were respected. Study participants with intestinal parasites or low hemoglobin concentration were linked to the nearby health facility.

## Results

A total of 5045 study participants was included giving for the response rate of 93.1%, 286 study participants did not volunteer to participate and 87 study participants were excluded due to incomplete data. The mean age of the study participants was 28 years (standard deviation [SD] ±14 years), 18 year was the youngest age of study participants, 1681 of the study participants was TB patients and 3364 of the study participants were TB free residents. Female constitute 53.9% (2721) of the study participants, 26% of the study participants were from the urban area, 90% of the study participants were Amhara by ethnicity and 96.7% of the study participants were Orthodox Christian believers.

### Profile of TB patients

A total of 1681 TB patients were included giving for the response rate of 93%. The mean age of the study participants was 27.78 years (SD ±13.98 years), 42.3% of TB patients were from the urban area and 76.8% of TB patients were pulmonary TB patients (Table 1).

**Table 1:** Profile of TB patients (*n* = 1681)

*S* ^*a*^	*Population profile*		*Frequency*	*Percentage*	*Normal BMI*		*Underweight*	
					*Frequency*	*Percentage*	*Frequency*	*Percentage*
1.	Sex	Male	1162	69.1	527	31.4	633	37.8
		Female	519	30.9	193	11.3	327	19.4
2.	Residence	Urban	711	42.3	218	13	493	29.3
		Rural	970	57.7	502	29.9	468	27.8
3.	Site of infection	Pulmonary tuberculosis	1297	76.8	556	33.1	735	43.7
		Extra-pulmonary TB	390	23.2	164	9.8	226	13.4
4.	HIV status	Positive	595	35.2	307	18.3	285	17
		Negative	1089	64.8	339	20.2	676	40.2
5.	Income in birr^b^	< 1000	1170	69.6	463	27.5	707	42.1
		1000–2000	316	18.8	186	11.1	130	7.7
		≥2001	195	11.6	71	4.2	124	7.4
6.	Occupation	Farmer	835	49.7	407	24.2	428	25.5
		Others	846	50.3	313	18.6	533	31.7
7.	Family size	≤4	648	38.5	499	29.7	149	8.9
		> 4	1033	61.5	221	13.1	812	48.3
8.	Marital status	Single	694	41.3	278	16.5	416	24.7
		Married	966	57.5	427	25.4	539	32.1
		Divorced	18	1.1	14	0.8	4	0.2
		Widowed	3	0.2	1	0.1	2	0.1
9.	Anemia	Present	1488	88.5	654	38.9	834	49.6
		Absent	193	11.5	66	3.9	127	7.6
10.	Age	< 25	1350	80.3	513	30.5	837	49.8
		≥25	331	19.7	207	12.3	124	7.4
11.	Intestinal parasitic infection	*Ascaris lumbricoides*	409	24.3	82	4.9	327	19.5
		*Hookworm*	282	16.8	112	6.7	170	10.1
		*Strongloid stercolaris*	75	4.5	19	1.1	56	3.3
		Others	127	7.6	22	1.3	105	6.2
		Not infected	788	46.9	485	28.9	303	18

^a^*SN* serial number

^b^1 US dollar =23 birr

The prevalence of underweight among TB patients was 57.17% (95% CI: 54.80 -59.54%), 88.52% of TB patients were anemic (95% CI: 86.99 - 90.04%) and 48.25% (718) of anemia was iron deficiency anemia (Table 2).

**Table 2:** The type of anemia among TB patients (*n* = 1488)

			Mean corpuscular volume (MCV)			Total	Malnutrition			
			Normocytic	Microcytic	Macrocytic		Norma		Underweight	
						No.	Frequency	Percentage	Frequency	Percentage
Mean corpuscular hemoglobin concentration (MCHC)	Normochromic		672	15	6	693	282	19	411	27.6
	Hypochromic		15	718	4	737	353	23.7	384	25.8
	Hyperchromic		14	12	32	58	19	1.3	39	2.6
	Total	No.	701	745	42	1488				
		%	47.11	50.07	2.82	100				

After adjusting for type of tuberculosis, sex, residence, educational status, intestinal parasitic infection, alcohol intake, age, HIV infection, family size, belief in avoiding a certain type of foods, income, smoking, and occupation; the following results were obtained: The odds of malnutrition among extra-pulmonary TB patients were 47% higher than pulmonary TB patients (AOR 0.68: [95% CI; 0.49–0.94]. In female TB patients, the odds of malnutrition were 2.56 higher than male. (AOR: 0.39 [95% CI; 0.25–0.56]). The odds of malnutrition among TB patients were 3.84 folds higher in the urban areas than the rural area (AOR 3.84 [95% CI: 2.74–5.54]). The odds of malnutrition were seven-folds higher among intestinal parasites positive TB patients than intestinal parasite negative TB patients (AOR 7: [95% CI: 5.2–9.95]). Problematic alcohol use increases the odds of malnutrition by 1.52 folds (AOR 1.52: [95% CI: 1.17–2.13]). Anemic TB patients had 3.23 folds higher risk of malnutrition than non-anemic TB patients (AOR 3.23: [95% CI; 1.89–5.51]). The odds of malnutrition were 3.23 higher in TB patients whose age was greater than 25 years (AOR 0.31 [95% CI; 0.21–0.46]). The odds of malnutrition were 1.96 folds higher among HIV positive TB patients than HIV negative TB patients (AOR 1.96: [95% CI; 1.47–2.7]). The odds of malnutrition were 15.75 folds higher among TB patients with high family size (AOR 15.75: [95% CI; 11.58–21.42]). Believe in avoiding certain types of food increase the odds of malnutrition by 3.19 folds (AOR 3.19: [95% CI: 2.37–4.31]). (Table 3).

**Table 3:** Malnutrition predictors in TB patients (*n* = 1681)

Variables		Malnutrition		COR^a^ [95% CI]	AOR^b^ [95% CI]	*P*-value
		Underweight	Normal BMI			
Type of TB	Pulmonary TB	735	556	0.96 [0.76–1.21]	0.68[0.49–0.94]	0.02
	Extra-pulmonary TB	226	164	Reference	Reference	
Sex	Male	635	527	0.71[0.58–0.88]	0.39 [0.25–0.56]	< 0.01
	Female	326	193	Reference	Reference	
Residence	Urban	493	218	2.43 [1.98–2.98]	3.84 [2.74–5.54]	< 0.01
	Rural	468	502	Reference	Reference	
Educational status	Literate	756	544	1.19 [0.95–1.5]	0.72 [0.52–1.02]	0.06
	Illiterate	205	176	Reference	Reference	
Intestinal parasitic infection	Present	658	235	4.48 [3.64–5.51]	7 [5.2–9.95]	< 0.01
	Absent	303	485	Reference	Reference	
Alcohol intake	Yes	430	277	1.29 [1.06–1.58]	1.52 [1.17–2.13]	< 0.01
	No	531	443	Reference	Reference	
Anemia	Present	834	654	0.66 [0.48–0.91]	3.23 [1.89–5.51]	< 0.01
	Absent	127	66			
Age	≥25	124	207	0.37 [0.29–0.47]	0.31 [0.21–0.46]	< 0.01
	< 25	837	513	Reference	Reference	
HIV infection	Positive	285	307	0.57 [0.46–0.70]	1.96 [1.47–2.7]	< 0.01
	Negative	676	413			
Family size	> 4	812	221	12.30 [9.65–15.69]	15.75 [11.58–21.42]	< 0.01
	≤4	149	499			
Believe in avoiding a certain type of food	Yes	588	209	3.85 [3.13–4.74]	3.19 [2.37–4.31]	< 0.01
	No	373	511			

^a^*COR* crude odds ratio

^b^*AOR* adjusted odds ratio

### Profile of TB free study participants

A total of 3364 TB free study participants was included giving for the response rate of 93.13%. The mean age of the study participants was 28.3 years (SD ±14.03 years). Female constitute 65.5% of the study participants, and 81.8% of the study participants were from the rural areas (Table 4).

**Table 4:** Profile of TB free study participants (*n* = 3364)

SN	Population profile		Frequency	Percentage	Normal BMI		Underweight	
					Frequency	Percentage	Frequency	Percentage
1.	Sex	Male	1162	34.5	851	25.3	331	9.2
		Female	2202	65.5	1727	51.3	475	14.1
2.	Residence	Urban	611	18.2	451	13.4	160	4.8
		Rural	2753	81.8	2127	63.2	626	18.6
3.	HIV status	Positive	31	0.9	15	0.4	16	0.5
		Negative	3333	99.1	2563	76.2	770	22.9
4.	Income in birr	< 1000	2558	76	2000	59.5	558	16.6
		1000–2000	316	9.4	227	6.7	89	2.6
		≥2001	490	14.56	351	10.43	139	4.13
5.	Occupation	Farmer	507	15.1	401	11.9	106	3.2
		Others	2857	84.9	2177	64.7	680	20.2
6.	Smoking	Yes	140	4.16	119	3.5	21	0.6
		No	3224	95.84	2459	73.1	765	22.7
7.	Family size	≤4	151	4.5	115	3.4	36	1.1
		> 4	3243	95.5	2463	73.2	750	22.3
8.	Marital status	Single	1474	43.8	1169	34.8	305	9.1
		Married	1854	55.1	1379	41	475	14.1
		Divorced	33	1	27	0.8	6	0.2
		Widowed	3	0.1	3	0.1	0	0
9.	Anemia	Present	1742	51.8	1475	43.8	267	7.9
		Absent	1642	48.2	1103	32.8	519	15.4
10.	Age in years	< 25	2622	77.9	1984	59	638	19
		≥25	742	22.1	594	17.7	148	4.4
11.	Intestinal parasitic infection	Ascaris Lumbricoides	530	15.8	331	9.8	199	5.9
		Hookworm	401	11.9	268	8	133	4
		Strongyloides stercolaris	99	2.9	74	2.2	25	0.7
		Others	433	12.9	319	9.5	114	2.7
		Not infected	1901	56.5	1586	47.1	315	9.4

### Malnutrition in the TB free residents

The prevalence of malnutrition (underweight) among TB free residents was 23.37% (95% CI: 21.93–24.80). The prevalence of anemia was 51.78% (95% CI: 50.09–53.47%). The predominant type of anemia was Normochromic Normocytic accounting for 63.32%, followed by Hypochromic Microcytic anemia 32.72%. (Table 5).

**Table 5:** The type of anemia among TB free residents (*n* = 1742)

		MCV			Total	Normal		Underweight	
						Frequency	Percentage	Frequency	Percentage
		Normocytic	Microcytic	Macrocytic					
MCHC	Normochromic	1103	11	6	1120	853	49	267	15.3
	Hypochromic	2	570	4	576	576	33.1	0	0
	Hyperchromic	2	12	32	46	46	2.6	0	0
	Total	1107	293	42	1742				

After adjusting for sex, residence, educational status, intestinal parasitic infection, alcohol intake, age, HIV infection, family size, believes in avoiding a certain type of food, income, smoking, and occupation; the following results were obtained:

The odds of malnutrition were 2.5 folds higher in male than female (AOR 2.5: [95% CI; 1.72–3.63]). Intestinal parasitic infection increases the odds of malnutrition by 3 folds higher (AOR 3.07: [95% CI; 2.38–3.96]). The odds of malnutrition were 7.82 folds higher among anemic residents (AOR 7.82: [95% CI; 5.74–10.63]) than non-anemic residents. The odds of malnutrition among study participants whose age was ≥25 years was 27% lower (AOR 0.73 [95% CI; 0.59–0.91]). HIV infection increases the odds of malnutrition by 4.51 folds than HIV free residents (AOR 4.5:1 [95% CI; 2.13–9.53]). (Table 6).

**Table 6:** Predictors of malnutrition among TB free residents (*n* = 3364)

Variables		Malnutrition		COR [95% CI]	AOR [95% CI]	*P*-value
		Underweight	Normal BMI			
Smoking	Yes	21	119	0.57 [0.34–0.93]	0.64 [0.39–1.05]	0.076
	No	765	2459			
Sex	Male	311	851	1.33 [1.12–1.57]	2.5[1.72–3.63]	< 0.01
	Female	475	1727			
Residence	Urban	160	451	1.21 [0.98–1.48]	0.89 [0.71–1.13]	0.34
	Rural	626	2127			
Income	< 2000 birr	734	2442	0.79 [0.56–1.11]	1.434 [1.00–2.06]	0.06
	≥2000 birr	52	136			
Intestinal parasitic infection	Present	471	992	2.39 [2.02–2.82]	3.07 [2.38–3.96]	< 0.01
	Absent	315	1586			
Alcohol intake	Yes	251	878	0.91 [0.76–1.08]	1.07 [0.88–1.29]	0.53
	No	535	1700			
Anemia	Present	267	1475	0.38 [0.32–0.46]	7.82 [5.74–10.63]	< 0.01
	Absent	519	1103			
Age	≥25	148	594	0.77 [0.63–0.95]	0.73 [0.59–0.91]	< 0.01
	< 25	638	1984			
HIV infection	Positive	16	15	3.55 [1.66–7.61]	4.51 [2.13–9.53]	< 0.01
	Negative	770	2563			
Family size	≤4	36	115	1.03 [0.69–1.53]	0.96 [0.63–1.43]	0.81
	> 4	750	2463			
Believe in avoiding a certain type of food	Yes	447	1283	1.33 [1.13–1.57]	0.87 [0.73–1.05]	0.14
	No	339	1295			

## Discussion

The prevalence of underweight among TB patients was 57.17%; the prevalence of underweight among TB free residents was 23.37%. This proportion was statistically significant (X^2^ 565.8, *P*-value < 0.01). This finding indicates that 33.8% of excess malnutrition was observed as a result of TB. This is because TB infection increases the anabolic process and consumes additional energy [37], additionally, TB infection manifests with a reduction in appetite, nutrient malabsorption, finally increasing the risk of underweight [34].

The prevalence of anemia among TB patients was 88.52%; the prevalence of anemia among TB free residents was 51.78%. TB patients had a 36.74% excess burden of anemia as compared to the residents. This result was statistically significant at X^2^ 656.7 *p*-value < 0.01. This is due to the effect of TB on red blood cell production like decreasing the erythrocyte lifespan, poor erythrocyte iron incorporation, and decreased sensitivity to our supply of erythropoietin [38].

The odds of malnutrition among extra-pulmonary TB patients were 47% higher than pulmonary TB patients. This finding was in line with finding from Nepal [39]. This is due to the reason that the diagnosis of extra-pulmonary TB is not easy as compared to pulmonary TB and patients were not early detected for the intervention [40]. The odds of malnutrition were 2.5 times higher in female TB patients than male TB patients. This finding agrees with finding from Ghana [20]. This is due to the reason that the severity of TB was more virulent in female [41]. Additionally, Male could have better access to food compared to the female who might need to prioritize their families, especially their children.

The odds of malnutrition among TB patients were 3.84 folds higher in the urban areas than the rural. This finding disagrees with finding from India [42]. This is due to the reason that most urban residents in Ethiopia were living in the overcrowded condition [43].

The odds of malnutrition were 7 folds higher among intestinal parasites infected TB patients than intestinal free TB patients. This finding agrees with finding from Butajira [44]. This is because intestinal parasites decrease the food intake, interfere with the absorption of nutrients and they share the host nutrients [45].

TB patients with problematic alcohol use had 1.52 fold higher risk of becoming malnourished than non-alcoholic TB patients. This finding agrees with research finding from Tanzania [46]. This is due to the reason that alcoholic patients eat poorly, had poor digestion, storage, use, and excretion of nutrients [47].

The odds of malnutrition among anemic TB patients were 3.23 folds higher than non-anemic TB patients. This finding agrees with finding from Brazil [48]. This is due to the effect of red blood cells in the transportation of nutrients and minerals [49].

The odds of malnutrition were 3.23 folds higher among TB patients whose age was greater than 25 years. This finding agrees with finding from Ghana [33]. This is due to the reason that the risks of co-morbid illness like non-communicable diseases were higher in old age [50].

The odds of malnutrition were 1.96 folds higher among HIV positive TB patients than HIV negative TB patients. This finding agrees with finding from Tanzania [46]. This is due to the reason that HIV positive patients were not economically productive so that they can’t get the access to the different variety of foods [51], HIV positive patients have a poor appetite, poor absorption of nutrients [52, 53].

The odds of malnutrition were 15.75 folds higher among TB patients with family size greater than 4. This finding agrees with finding from India [20]. This is because high family size decreases the household income leading to the low dietary intake of household members [54].

Believe in avoiding a certain type of foods (mostly animal products) increase the odds of malnutrition by 3.19 folds. This finding was in line with finding from Ghana [55]. This is due to the reason that patients will not take important nutrients due to their behavior of avoiding that type of food [56].

Family size and believe in avoiding a certain type of food were not the predictors of malnutrition among the tuberculosis-free residents, but these variables were the predictors of malnutrition among TB patients. These show that TB patients are very susceptible to malnutrition even the very distal reason for malnutrition in the community became a proximal reason for TB patients.

The limitation of the study might be related to the cross-sectional nature of the study, which makes difficult to find whether the exposure precedes the outcome. However, this limitation works only for modifiable variables thought time. Since we used patient interview methods, recall bias might occur, however, we included only newly diagnosed TB patients which significantly drop the recall bias.

## Conclusion

A very high proportion of TB patients were malnourished. TB patients were highly susceptible to malnutrition and even a very distal factor for malnutrition became proximal for TB patients. The nutritional status of TB patients was affected by the site of infection, sex, residence, intestinal parasite infection, alcohol.

### Recommendation

The DOTS for TB intervention should consider incorporating more nutritional support for the patients. Because a significant proportion of TB patients were malnourished, they can’t afford access to nutritionally rich foods and administering the anti-TB drugs alone will decrease the treatment success rate. Also, the guideline should consider iron supplementation and deworming as part of the TB treatment.

## Data Availability

The datasets used and/or analyzed during the current study are available from the corresponding author on reasonable request.

## Abbreviations

AOR: Adjusted odds ratio
BMI: Body Mass Index
CI: Confidence Interval
COR: crude odds ratio
DOTS: Directly Observed Treatment Strategy
*H.nana*: Hemnolephus Nana
HC: Health Center
IT: Information Technology
MCHC: Mean corpuscular hemoglobin concentration
MCV: Mean corpuscular volume
MDR-TB: Multidrug Resistance Tuberculosis
ML: Milliliters
OPD: Out Patient Department
OR: Odds Ratio
RPM: Revolution per Minute
SAF: Sodium Acetate Acetic Acid Formulation
SD: Standard Deviation
SPSS: Statistical Software for Social Science
TB: TB
USA: United State of America
WHO: World Health Organization
